# Feshbach resonances in the F + H_2_O → HF + OH reaction

**DOI:** 10.1038/s41467-019-14097-y

**Published:** 2020-01-13

**Authors:** Xiaoren Zhang, Lulu Li, Jun Chen, Shu Liu, Dong H. Zhang

**Affiliations:** 1 State Key Laboratory of Molecular Reaction Dynamics, Dalian Institute of Chemical Physics, Chinese Academy of Sciences, Dalian, 116023 China; 2 University of Chinese Academy of Sciences, Beijing, 100049 China; 3 University of Science and Technology of China, Hefei, 230026 China; 4 College of Chemistry and Chemical Engineering, Xiamen University, Xiamen, 361005 China

**Keywords:** Reaction kinetics and dynamics, Reaction mechanisms

## Abstract

Transiently trapped quantum states along the reaction coordinate in the transition-state region of a chemical reaction are normally called Feshbach resonances or dynamical resonances. Feshbach resonances trapped in the HF–OH interaction well have been discovered in an earlier photodetchment study of FH_2_O^−^; however, it is not clear whether these resonances are accessible by the F + H_2_O reaction. Here we report an accurate state-to-state quantum dynamics study of the F + H_2_O → HF + OH reaction on an accurate newly constructed potential energy surface. Pronounced oscillatory structures are observed in the total reaction probabilities, in particular at collision energies below 0.2 eV. Detailed analysis reveals that these oscillating structures originate from the Feshbach resonance states trapped in the hydrogen bond well on the HF(*v*′ = 2)-OH vibrationally adiabatic potentials, producing mainly HF(*v*′ = 1) product. Therefore, the resonances observed in the photodetchment study of FH_2_O^−^ are accessible to the reaction.

## Abstract (web summary)

Feshbach resonances are transiently trapped states along a reaction coordinate, providing a probe to the reaction’s potential energy surface (PES) but difficult to analyze in polyatomic systems. Here the authors identify Feshbach resonances in a reacting 4-atom system by state-to-state quantum dynamics using a full-dimensional PES.

## Introduction

Feshbach resonances of a chemical reaction are transiently trapped quantum states along the reaction coordinate in the transition-state region. They not only have a profound influence on both the rate and product distribution, but also provide sensitive probes to the potential energy surface (PES) of the reaction. Over the past decades, great efforts have been devoted to detecting resonances in chemical reactions and to studying their structures and dynamics^1–3^. Thanks to the close interaction between theory and experiment, an accurate physical picture of dynamical resonances in the F + H_2_ (HD) → HF + H(D) reaction has been well established^4–12^. Detailed quantum dynamics studies on increasingly more accurate PESs uncovered that the interaction between H(D) and HF in the post-barrier region softens the HF bond and creates a peculiar well on the HF(*v*′ = 3)-H(D) vibrationally adiabatic potential (VAP), which supports resonance states producing mainly HF(*v*′ = 2) product^8,9^.

Similar to the F + H_2_ system, the F + H_2_O → HF + OH reaction is highly exothermic (Δ*H*^0^ = −17.6 kcal/mol) and has a low and “early” barrier on the ground electronic state. There is a relatively deep van der Waals (vdw) well (~3 kcal/mol) in the entrance channel and a hydrogen bond (HB) well (~6 kcal/mol) in the exit channel. The reaction is of great importance in atmospheric^13^ and astrochemistry^14^, and has attracted a large number of experimental and theoretical studies. The reaction dynamics was first investigated experimentally by Setser and colleagues^15,16^, who found the HF product is vibrationally excited and rotationally cold. After that, Nesbitt and colleagues^17,18^ observed a highly nascent vibrational population inversion for the HF(*v* = 1) product. Theoretically, as the number of the electrons is relatively small, this system represents an attractive prototype for the electronic structure and reaction dynamics calculations. Guo and colleagues^19^ developed a full-dimensional global PES for the F + H_2_O system using a multireference configuration interaction (MRCI) method and applied a standard quasi-classical trajectory (QCT) study. In agreement with experiment, the product state distribution is dominated by HF vibrational excited states. The subsequent QCT and quantum mechanical (QM) calculations found that the pre-reaction vdw well enhance reactivity at low energies due to stereodynamics^20^ and the vibrational excitation of H_2_O is much more efficient at promoting the reactivity than the translational energy^21^. On the other hand, Guo and colleagues^22–24^ also found the coupled-cluster method with higher excitations than singles and doubles gives a better description than the MRCI method for the barrier height. They scaled the original MRCI PES with an external correlation scaling method and provided both kinetic and dynamical data for the reaction on the scaled-MRCI PESs^22–24^.

In 2014, Otto et al. carried out a joint experimental and theoretical study on the FH_2_O^−^ photodetachment dynamics. Both the measured and calculated photoelectron spectra of the anion were found to possess both broad and sharp features^25–28^. The former were attributed to short-lived reactive Feshbach resonances and the latter to long-lived nonreactive Feshbach resonances, both supported by product well^28^. At about the same time, Zhao and Guo^29^ reported a state-to-state quantum dynamics study on the reaction by using transition-state wave packet method for collision energy up to 2 kcal/mol on the scaled-MRCI PES. Strong oscillatory structures were observed for the *J* = 0 quantum reaction probabilities as a function of collision energy. However, they attributed these oscillations to tunneling resonances supported by the pre-reaction vdw well^29^. Therefore, the following questions clearly emerge: Can the reactive Feshbach resonances observed in the photodetachmment study be observed in the F + H_2_O reaction? And how these resonances affect the reaction?

Here we report an accurate state-to-state quantum scattering study of the title reaction on an accurate newly constructed neural network (NN) PES by using the time-dependent wave packet (TDWP) method^30,31^. The total reaction probabilities for collision energy below 0.2 eV exhibit many pronounced oscillatory structures especially. Analysis of scattering wave functions and VAPs reveal that these oscillating structures originate from the Feshbach resonance states trapped in the HF(*v*′ = 2)-OH VAP well, producing mainly the HF(*v*′ = 1) product. This suggests the Feshbach resonances observed in the photodetachment study can be accessed by the neutral reaction.

## Results

### Total reaction probabilities and the product-state distributions

**Fig. 1:**
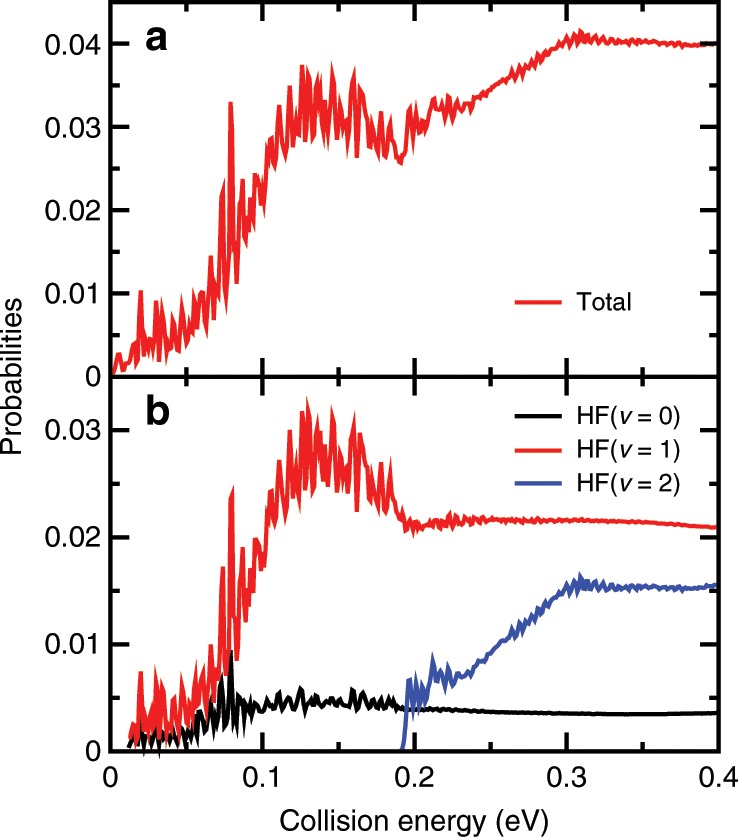
Reaction probabilities for the ground rovibrational initial state. **a** Total and **b** product HF vibrational state-resolved reaction probabilities as a function of collision energy.

The converged total reaction probabilities for the total angular momentum *J* = 0 in Fig. 1a exhibit many sharp and narrow oscillatory structures especially for collision energy below 0.2 eV. Overall, the reaction probabilities on the present PES resemble those obtained on the scaled-MRCI permutation invariant polynomial NN PES with spin–orbit (SO) corrections^24^ rather well, except the oscillatory structures and a shift of energy by ~0.015 eV apparently due to different barrier heights for these two PES, but larger than those obtained on the original MRCI PES^20^ (Supplementary Fig. 3). Figure 1b shows the product HF vibrational state-resolved reaction probabilities as a function of collision energy. The HF(*v* = 1) state has the largest population in the entire energy region considered here, indicating an inverted HF vibrational state distribution. Before the opening of the HF(*v* = 2) channel, the HF(*v* = 0) and HF(*v* = 1) probabilities have rich oscillating structures. The HF(*v* = 2) channel opens at the collision energy of 0.191 eV and also has some small oscillating structures in probability. Meanwhile the HF(*v* = 0) and HF(*v* = 1) probabilities become rather smooth at high collision energies.

**Fig. 2:**
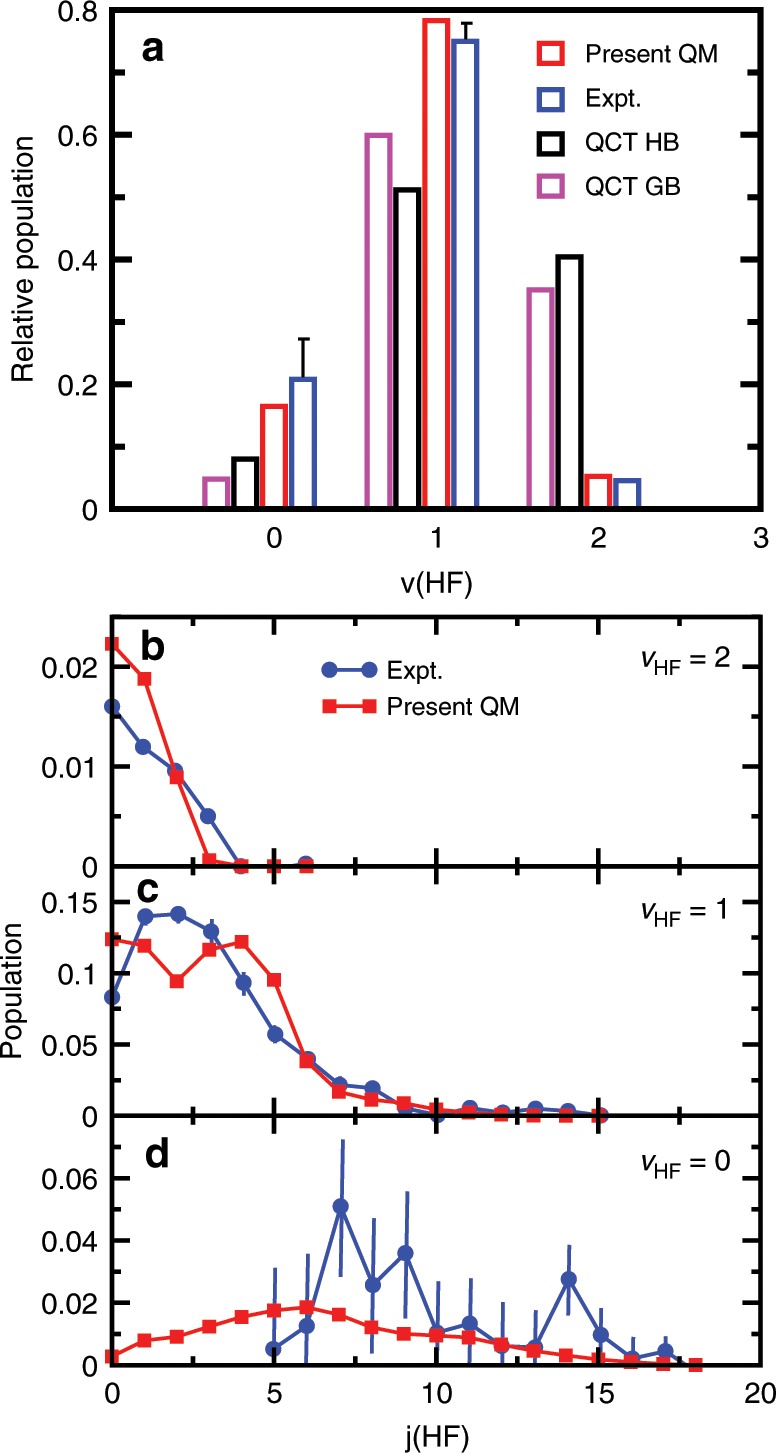
Product-state distributions. The normalized HF vibrational state distributions (**a**) and rotational state distributions for each *v* (**b**–**d**) at the collision energy of 0.234 eV (5.4 kcal/mol), in comparison with the experimental values with error bars (SD)^16^ and the quasi-classical trajectory results on the permutation invariant polynomial potential energy suface^22^.

To compare with the experimental and previous QCT results, we calculated the rovibrational state-resolved integral cross-sections based on the reaction probabilities for total angular momentum *J* = 0 by using the J-shifting approximation, i.e., *P*^*J*>0^(*E*) = *P*^*J*=0^(*E* − Δ*E*), where Δ*E* = B*J(*J* + 1). The rotational constant is approximately calculated by B* = (2μR)^−1^ (R*)^2^ with R* being the length of the scattering coordinate at the transition-state^32,33^. Figure 2a shows the normalized vibrational populations and Fig. 2b–d show the normalized rotational populations for each *v*′(*v*′ = 1–3), of product HF at the collision energy of 0.234 eV (5.4 kcal/mol). The present QM vibrational state distributions agree quite well with the experiment, while the previous QCT results overestimate the population for *v*′ = 2. The calculated rotational state distributions, which changes from a broad one with a maximum at *j* = 6 for *v*′ = 0 to a narrow one with a maximum at *j* = 0 for *v*′ = 2, are also consistent with the experiment.

### Analysis of scattering wave functions and VAP

**Fig. 3:**
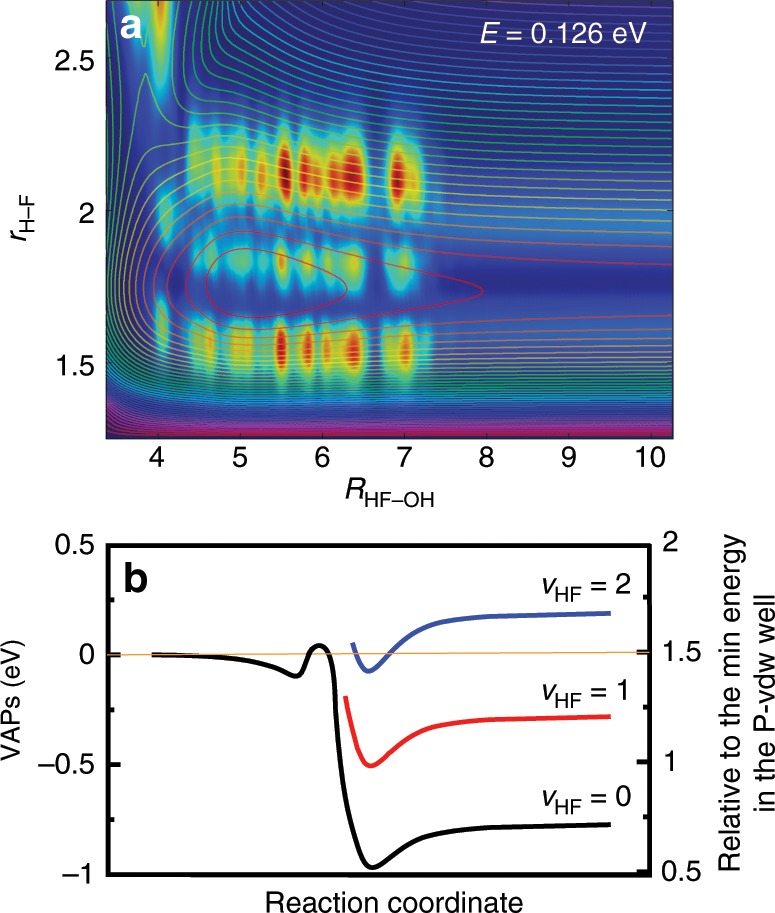
Scattering wave functions and vibrational adiabatic potentials analyses. **a** Reactive scattering wave functions for the title reaction in the two Jacobi coordinates *R*(HF–OH) and *r*(H-F) with other coordinates integrated at the collision energies of 0.126 eV. The contour lines are the corresponding two-dimensional potential energy suface. **b** The calculated vibrational adiabatic potentials for different vibrational states of HF.

To investigate the dynamical original of the oscillatory structures, we extract the scattering wave function at the collision energy of 0.126 eV for *J* = 0. Inspection of the two-dimensional contour of the wave function in the product region shown in Fig. 3a reveals there exist two nodes along the H–F coordinate (correlating to the HF product) inside the HB well, but only one node for the outgoing wave function with a sudden decrease in amplitude. This sudden change of nodal structure and amplitude in wave function is the characteristic feature of a Feshbach resonance, indicating the resonance is trapped in HF(*v*′ = 2) vibrational adiabatic well and produces mainly HF(*v*′ = 1) product.

To further confirm the mechanism of the resonance, we calculated the VAP for HF(*v*′ = 0–3) in the product region. As shown in Fig. 3b, the calculated VAP for all these vibrational states of HF have HB wells, which may support resonance states for the system. The HF(*v*′ = 2)-OH VAP has an asymptotic value of 0.193 eV measured from the ground state of the reactants and has a well with a well depth of 0.26 eV at a separation distance between HF and OH equal to 5.0 *a*_0_, i.e., the bottom of well is lower than the ground state of the reactants by 0.067 eV. The calculations show that the zero-point energy of the HF–OH in the well is also lower than the ground state of reactant. This means the F + H_2_O reaction can access to the Feshbach resonances supported by HF(*v*′ = 2)-OH VAP well even with zero collision energy and the oscillating structures on the HF(*v*′ = 0) and HF(*v*′ = 1) probabilities in Fig. 1b originate from these Feshbach resonance states. In addition, the small oscillating structures on the HF(*v*′ = 2) probabilities at the collision energy above 0.191 eV apparently originate from the bending excited HF(*v*′ = 2)-OH VAP supported resonance states.

As can be seen from Fig. 3b, the minimum positions for the HF(*v*′)-OH VAP wells for *v*′ = 0-2 all locate *R*~5.0 a_0_, whereas the well depth increases with *v*′, from 0.19 eV for *v*′ = 0, to 0.22 eV for *v*′ = 1, and 0.26 eV for *v*′ = 2. Therefore, these VAP wells are HB wells for the HF and OH products mainly through dipole–dipole interaction. With increase of the HF vibrational state, HF diploe moment increases, resulting in a deeper well depth. The Feshbash resonances for the F + H_2_O reaction are trapped in HF(*v*′ = 2)-OH HB well.

It is well known that in the F + H_2_ reaction, the interaction between H and HF in the transition-state region softens the HF bond, manifesting a larger anharmonicity for HF vibration. This anharmonicity considerably lowers the energy level for the HF(*v*′ = 3) vibrationally excited state, resulting in a peculiar well on the H-HF(*v*′ =  3) VAP right after the barrier position. This well is substantially deeper and locates much closer to barrier than the H-HF(*v*′ = 0–2) VAP wells, which originate from the vdw interaction between H and HF. Therefore, the resonance supporting HF(*v*′ = 2)-OH well in the F + H_2_O reaction is very different in nature from the H-HF(*v*′ = 3) VAP well, which support resonance states in the F + H_2_(*v* = 0) reaction^8,34,35^.

### Relation between FH_2_O- photoelectron spectra and neutral reaction probabilities

**Fig. 4:**
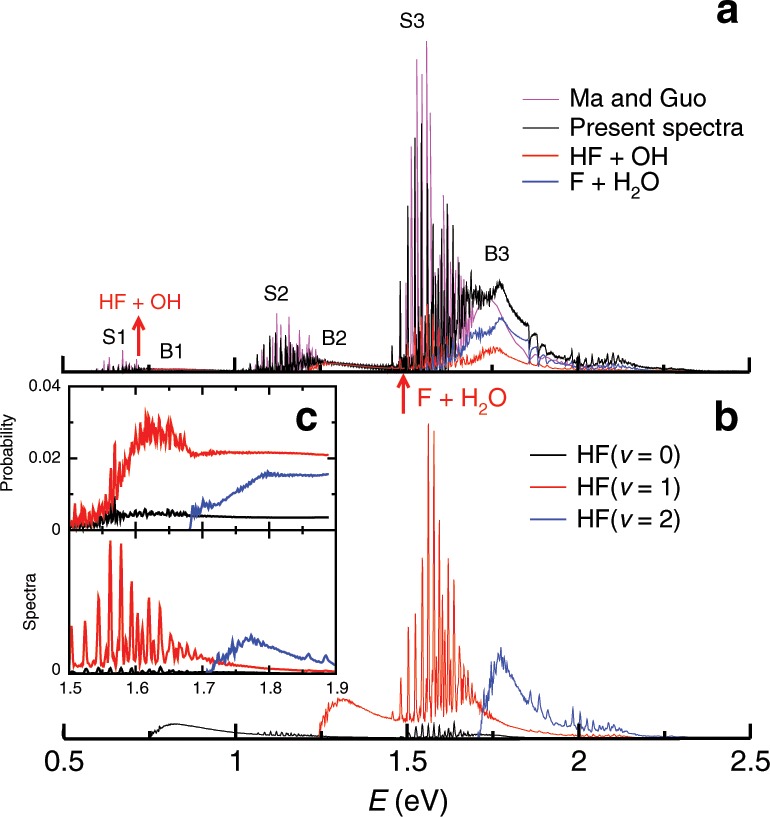
Photoelectron spectra of the FH_2_O^−^ system. **a** Theoretical photoelectron spectra and reactive flux spectra of the FH2O^−^ anion on the present neural network potential energy suface, in comparison with the spectra of Ma and Guo28. E is relative to the minimum energy of the neutral PES, i.e., the static energy of the bottom of the product hydrogen bond well. The red arrows indicate the zero-point energy of the HF + OH asymptote (0.718 eV) and F + H_2_O (1.488 eV) asymptote, respectively. **b** The HF vibrational state-resolved reactive flux spectra to HF + OH. **c** The comparison between the HF vibrational state-resolved reaction probabilities of the neutral reaction and the reactive flux spectra at the energy of 1.5–1.9 eV.

The photodetachment of the FH_2_O^−^ anion is also investigated quantum mechanically in full-dimensions on the new accurate NN PES. The anion PES is a newly constructed one by this group based on 11,936 CCSD(T)-F12a/(O and F: cc-pCVTZ-F12, H: cc-pVTZ-F12)^36^ ab initio energies with fundamental invariants NN (FI-NN) method^37^, yielding a root-mean-square-error of 0.043 meV. The ground rovibrational eigenstate on the anion PES is calculated and vertically projected onto the neutral PES for a continuous propagation using the diatom–diatom Jacobi coordinates. Figure 4a presents the total photoelectron spectra of the FH_2_O^−^ system, showing groups of sharp peaks, denoted as S1~S3, accompanied with broad features in the high-energy wings (B1~B3). The present spectra are very similar to that of Ma and Guo^28^, except for the position of the B3 peak, which comes from the difference of the two neutral PESs. Figure 4a also shows the reactive flux spectra to both the HF + OH and F + H_2_O products. As can be seen, F + H_2_O channel becomes dominant after opening. Figure 4b shows the product HF vibrational state-resolved reactive flux spectra to HF + OH. According to the VAP in Fig. 3b, the sharp peaks correspond to Feshbach resonance states trapped in the well, which can only decay into lower HF vibrational states by vibrational predissociation, whereas the broad features correspond to direct dissociation products. To compare with the probabilities of the neutral reaction, we present the spectra only for energy between 1.5 and 1.9 eV in Fig. 4c. As can be seen, the reaction probabilities resemble the spectrum very well, except the oscillatory structures in the reaction probabilities are much richer, indicating the resonance mechanism of the two processes is the same.

## Discussion

Therefore, the polyatomic F + H_2_O reaction also possesses Feshbach resonances trapped in VAP well in product region, similar to the triatomic F + H_2_ reaction. However, different from the F + H_2_ reaction in which the HF bond softening creates a peculiar well on the H-HF(*v*′ = 3) VAP, the VAP well for the F + H_2_O reaction is mainly HB in nature. Earlier photodetachment study of FH_2_O^−^ discovered Feshbach resonances trapped in the HF + OH interaction well. In this study, we unambiguously show that the Feshbach resonances observed in the photodetachment study actually can be accessed by the F + H_2_O reaction. More efforts, in particular more joint efforts between theory and experiment should be devoted to studying these Feshbach resonances in the reaction at a quantitative level of accuracy, and to pushing forward our understanding of Feshbach resonances from triatomic reactions to polyatomic reactions.

## Methods

### Potential energy surface

The PES used in the calculation is a full-dimensional global one constructed using NN method based on ~26,429 AE-UCCSD(T)-F12a/CVTZ-F12 ab initio energies. The correction terms considering the influence of a larger basis set as well as SO couplings were further implemented. With an overall fitting error of ~1.78 meV measured in terms of root-mean-square-error (Supplementary Table 1), the PES is expected to have a quantitative level of accuracy. The static barrier height for the PES is 1.922 kcal/mol (and raised to 2.262 kcal/mol with SO energy included), lower than the value of 3.838 kcal/mol for the MRCI PES^19^ and very close to the value of 1.919 suggested by Dawes and collegues^22^.

### Quantum dynamics calculations

Our state-to-state quantum calculation (*J* = 0) is carried out by using the product coordinates based^30,38^ scheme-based TDWP method with the coordinate systems shown in Supplementary Fig. 1. Test calculations reveal that the potential-averaged five-dimensional method with the nonreactive OH frozen at the ground vibrational state is of quantitative accuracy for the total reaction probabilities when compared with the full six-dimensional results as shown in Supplementary Fig. 2. The details of the PES and dynamics calculation are provided in Supplementary Information.

## Supplementary information

Supplementary Information

## Data Availability

The data that support the findings of this study are available from the corresponding author upon request.

## Code availability

The accurate NN PESs developed in this work are available from the corresponding authors upon request.
