# Supplementary material for: Feshbach resonances in the F + H2O → HF + OH reaction
Source: Nat Commun. 2020 Jan 13;11:223. doi: 10.1038/s41467-019-14097-y (PMC6957676; doi:10.1038/s41467-019-14097-y)
Supplement: Supplementary file 1 — Supplementary Information [file 41467_2019_14097_MOESM1_ESM.pdf]

Supplementary Information

Feshbach Resonances in the F + H_2O → HF + OH reaction

Zhang et al.

Supplementary Methods

A. Potential energy surface

The potential energy surface (PES) of F+H_2O system was constructed using the all-electron spin restricted explicitly correlated singles and doubles coupled-cluster approach (AE-CCSD(T)), together with optimized correlation consistent triple-zeta basis set including RI and MP2 auxiliary sets (cc-pCVTZ-F12)[1]. The spin-orbit coupling (SO) energies were computed with the internally contracted multi-reference configuration interaction method with Davidson correction (iMRCI+Q) and the basis set of aug-cc-pVTZ, using multi-configurational SCF reference functions with 15 electrons in 10 active orbitals, followed by the Breit-Pauli Hamiltomian[2]. All these calculations were performed with the MOLPRO 2012.1 package[3].

More than 24,000 points were used to construct the PES using the neural networks. These points, covering the asymptotic reactive channels of F+H_2O, HF+HO, as well as interaction region, were selected iteratively by an effective scheme[4] which was proposed for high dimensional PES constructions. The 6 bond lengths are used for the input layer. The asymptotic channels and interaction region were fitted segmentally to improve the fitting accuracy and efficiency, and connected with smooth switch functions. The switch functions for each part are defined from the bond distances between F and H atoms as (with H atoms sorted to satisfy $r_{\mathrm{F-H1}} \leq r_{\mathrm{F-H2}}$):

$$r_1 = \min(r_{\mathrm{F-O}}, r_{\mathrm{F-H1}}, r_{\mathrm{F-H2}})$$

$$r_2 = \min(r_{\mathrm{H2-F}}, r_{\mathrm{H1-O}}, r_{\mathrm{H1-H2}}, r_{\mathrm{F-O}})$$

$$w_{\mathrm{F+H2O}} = logsig[5.0(r_1 - 5.0)]$$

$$w_{\mathrm{HF+HO}} = (1 - w_{\mathrm{F+H2O}}) * logsig[5.0(r_2 - 5.0)]$$

$$w_{\mathrm{INT}} = 1 - w_{\mathrm{F+H2O}} - w_{\mathrm{HF+HO}}. \quad (1)$$

The final functional form of F+H_2O PES is written as:

$$V = V_{\mathrm{SO}} + V_{\mathrm{F+H2O}} w_{\mathrm{F+H2O}} + V_{\mathrm{HF+HO}} w_{\mathrm{HF+HO}} + V_{\mathrm{INT}} w_{\mathrm{INT}}. \quad (2)$$

The parameters of NN structures and fitting errors of each part are listed in Supplementary Table 1. Extensive trajectory and quantum scattering tests show that, with the overall fitting error of 1.78 meV, both the fitting procedure and the number of *ab initio* points are fully converged.

B. Coordinates and wavefunction representations for reactant and product arrangements

The coordinate systems and wavefunction representations for both atom-triatom and diatom-diatom scattering studies are outlined here. Supplementary Figure 1 shows the reagent Jacobi coordinates $(R, r_1, r_2, \theta_1, \theta_2, \varphi)$ for the F+H_2O atom-triatom arrangement, and product Jacobi coordinates $(R', r_1', r_2', \theta_1', \theta_2', \varphi')$ for the HF+OH diatom-diatom arrangement. As can be seen r_2 and r_2' share the same vector.

The system Hamiltonian in the reactant Jacobi coordinates for a given total angular momentum $\mathbf{J}_{\rm tot}$ can be written as

$$\hat{H} = -\frac{\hbar^2}{2\mu_R}\frac{\partial^2}{\partial R^2} + \frac{(\mathbf{J}_{\rm tot} - \mathbf{j}_{12})^2}{2\mu_R R^2} + \frac{\mathbf{j}_1^2}{2\mu_1 r_1^2} + \frac{\mathbf{j}_2^2}{2\mu_2 r_2^2} + V(R, r_1, r_2, \theta_1, \theta_2, \varphi)$$

$$+h_1(r_1) + h_2(r_2) \qquad (3)$$

where μ_R is the reduced mass of F and H_2O, μ_1 is the reduced mass of H and OH, μ_2 is the reduced mass of OH. $\mathbf{J}_{\rm tot}$ is the total angular momentum operator of the system; $\mathbf{j}_{12}$ is the rotational angular momentum operator of H_2O; $\mathbf{j}_2$ is the rotational angular momentum operator of OH, and $\mathbf{j}_1 = \mathbf{j}_{12} - \mathbf{j}_2$ is the orbital angular momentum operator of H_2O. The reference Hamiltonian $h_i(r_i)$ $(i = 1,2)$ is defined as

$$h_i(r_i) = -\frac{\hbar^2}{2\mu_i}\frac{\partial^2}{\partial r_i^2} + V_i(r_i) \qquad (4)$$

where $V_i(r_i)$ is a diatomic potential.

The time-dependent wave function can be expanded in terms of the translational basis of *R*, the vibrational basis $\phi_{v_i}(r_i)$, and the body-fixed (BF) rovibrational eigenfunction as

$$\Psi_{v_0 j_0 K_0}^{J_{tot} M \varepsilon}(\mathbf{R}, \mathbf{r}_1, \mathbf{r}_2, t) =$$

$$\sum_{nvjK} F_{nvjK, v_0 j_0 K_0}^{J_{tot} M \varepsilon}(t)\, u_n^{v_1}(R) \phi_{v_1}(r_1) \phi_{v_2}(r_2) Y_{jK}^{J_{tot} M \varepsilon}(\hat{R}, \hat{r}_1, \hat{r}_2). \qquad (5)$$

The BF total angular momentum eigenfunctions can be defined as

$$Y_{jK}^{J_{tot} M \varepsilon}(\hat{R}, \hat{r}_1, \hat{r}_2) = (1 + \delta_{\bar{K}0})^{-1/2} \sqrt{\frac{2J+1}{8\pi}} [\bar{D}_{M\bar{K}}^{J_{tot}}(\hat{R}) Y_{j_1 j_2}^{j_{12}\bar{K}}$$

$$+\varepsilon(-1)^{J_{tot}+j_1+j_2+j_{12}}\bar{D}^{J_{tot}}_{M-\bar{K}}(\hat{R})Y^{j_{12}-\bar{K}}_{j_1 j_2}] \quad (6)$$

where ε is the parity of the system. $\bar{D}^{J_{tot}}_{MK}(\hat{R})$ is the Wigner rotation matrix, depending on Euler angles which rotate the space-fixed frame onto the body-fixed frame and are the eigenfunctions of $\mathbf{J}^2_{tot}$. $Y^{j_{12}\bar{K}}_{j_1 j_2}(\hat{r}_1,\hat{r}_2)$ is the angular momentum eigenfunction of $\mathbf{j}_{12}$ defined as

$$Y^{j_{12}\bar{K}}_{j_1 j_2} = \sum_m \bar{D}^{j_{12}}_{Km}(\hat{r}_1)\sqrt{\frac{2j_1+1}{4\pi}} < j_2 m j_1 0 | j_{12} m > \mathcal{Y}_{j_2 m}(\hat{r}_2) \quad (7)$$

where $\mathcal{Y}_{j_2 m}$ are spherical harmonics. Note that the restriction $\varepsilon(-1)^{J_{tot}+j_1+j_2+j_{12}} = 1$ for K=0 partitions the whole rotational basis set into even and odd parities. Thus a K=0 initial state can only appear in one of these two parity blocks. For K>0, however, there is no such restriction, the basis set is the same for even and odd parities. Hence a K>0 initial state can appear in both parities.

For the the HF+OH arrangement, similarly to the discussion about F+H_2O arrangement, we have the same forms of equations, except the quantities with unprimed indices replaced by primed ones. The system Hamiltonian for the HF+OH arrangement can be written as

$$\hat{H} = -\frac{\hbar^2}{2\mu_{R'}}\frac{\partial^2}{\partial R'^2} + \frac{(\mathbf{J}_{\text{tot}} - \mathbf{j}'_{12})^2}{2\mu_{R'}R'^2} + \frac{\mathbf{j}'^2_1}{2\mu'_1 r'^2_1} + \frac{\mathbf{j}'^2_2}{2\mu'_1 r'^2_2} + V(R', r'_1, r'_2, \theta'_1, \theta'_2, \varphi')$$

$$+h_1(r'_1) + h_2(r'_2) \quad (8)$$

where $\mu_{R'}$ is the reduced mass of HF and OH, μ'_1 and μ'_2 are the reduced mass of HF and OH, $\mathbf{j}'_1$and $\mathbf{j}'_2$ are the rotational angular momentum operator of HF and OH, which coupled to form $\mathbf{j}'_{12}$.

The time-dependent wave function can be expanded as

$$\Psi^{J_{tot}M\varepsilon}_i(\mathbf{R}', \mathbf{r}'_1, \mathbf{r}'_2, t) =$$

$$\sum_{n'v'j'K'} F^{J_{tot}M\varepsilon}_{n'v'j'K',i}(t)\, u^{v'_1}_{n'}(R')\phi_{v'_1}(r'_1)\phi_{v'_2}(r'_2)Y^{J_{tot}M\varepsilon}_{j'K'}(\hat{R}', \hat{r}'_1, \hat{r}'_2). \quad (9)$$

It should be pointed out here that the functional forms for the product arrangement basis (primed) are different from the one for the reagent arrangement basis (unprimed).

The BF total angular momentum eigenfunctions can be defined as

$$Y_{j'K'}^{J_{tot}M\varepsilon}(\hat{R}',\hat{r}_1',\hat{r}_2') = (1+\delta_{K'0})^{-1/2}\sqrt{\frac{2J+1}{8\pi}}\,[\bar{D}_{M\bar{K}'}^{J_{tot}}(\hat{R}')Y_{j_1'j_2'}^{j_{12}'\bar{K}'}$$

$$+\varepsilon(-1)^{J_{tot}+j_1'+j_2'+j_{12}'}\bar{D}_{M-\bar{K}'}^{J_{tot}}(\hat{R}')Y_{j_1'j_2'}^{j_{12}'-\bar{K}'}]. \quad (10)$$

$Y_{j_1'j_2'}^{j_{12}'\bar{K}'}$ is the angular momentum eigenfunction of $\hat{j}_{12}'$

$$Y_{l'j'k'}^{J'K'}(\hat{r}',\hat{s}) = \sum_{m_1} < j_1'm_1j_2'j_{12}'\bar{K}' - m_1|j_{12}'\bar{K}' > \mathcal{Y}_{j_1'm_1}(\hat{r}_1')\mathcal{Y}_{j_2'\bar{K}'-m_1}(\hat{r}_2'). \quad (11)$$

C. PCB approach and numerical parameters

In the product coordinates based (PCB) approach[5,6] used here, we prepared an initial wave packet for H_2O in the initial ground rovibrational state in the reactant Jacobi coordinates, and propagated it for 17000 a.u. from the asymptotic region to R=6.0 bohrs. It is straightforward to carry out this propagation, because at that R distance, only inelastic scattering process occurs. A coordinate transformation was then carried out to transfer the whole wave packet from the reactant coordinates to the product coordinates. After a continuous propagation for additional 80000 a.u. in the product coordinates, which beyond the range of the hydrogen bond well and strong interaction between HF and OH species, the converged reactive flux and state-to-state information can be obtained.

The wavefunction is propagated using the split-operator propagator. An L-shaped wavefunction expansion for $R(R')$ and $r_1(r_1')$ was used to reduce the size of the basis set. We carried out state-to-state calculations for the total angular momentum $J_{\mathrm{tot}} = 0$ for collision energies up to 0.4 eV. The numerical parameters used in the wave packet propagation for the F+H_2O system are as the following. A total number of 700 sine basis functions covering a range from 2.0 to 44.0 bohrs were used for R. For the r_1 dimension, 8 basis functions were used in the range of [1.0,6.0] bohrs in the asymptotic region. The rotational basis functions was constrained by the parameters, $j_{1\mathrm{max}}$=30, $j_{2\mathrm{max}}$=28. The width of the prepared initial Gaussian wave packet was 0.18 bohr, and the central energy was E_0=0.2 eV.

For the HF+OH system, a total number of 392 sine basis functions covering a

range from 0.5 to 20.0 bohrs were used for R' with 200 grid points in the interaction region. For the r_1' dimension, 120 basis functions were used in the range of [1.0,12.0] bohrs in the interaction region, while 9 basis functions were used in the asymptotic region. The rotational basis functions was constrained by the parameters, $j'_{1\mathrm{max}}$=98, $j'_{2\mathrm{max}}$=28. A dividing surface is placed at R'=15.5 bohrs to extract *S*-matrix elements.

D. Test calculations of the potential-averaged five-dimensional method

Supplementary Figure 2 shows the full-dimensional (6D) total reaction probabilities as a function of collision energy with both OH bonds treated as reactive (in the interaction region, 70 basis functions were used in the range of [1.0,9.0] bohrs for r_1, 40 basis functions were used in the range of [1.0,7.0] bohrs for r_2, in comparison with the potential-averaged five-dimensional (PA5D) results. The PA5D probabilities are only slightly different from the full 6D ones, indicating the OH bond is a good spectator for the reaction, and can be fixed in its initial vibrational state. With two heavy atoms (F and O) involved and long-range dipole-dipole interactions in the exit channel, the computation is extremely expensive. To reduce the computational costs, the results in the main text are based on the PA5D calculations. Since there are two equivalent product channels in the reaction, the reaction probabilities should be multiplied by a factor of 2, if compared with QCT results.

	Number of points	NN structure	Fitting error (meV)
1. F + H_2O	4769	6-30-30-1	0.07
2. *vdw*(F…H_2O)	13933	6-50-50-1	1.96
3. HF+HO	19523	6-30-30-1	0.93
**In total**	**24427**	6-50-50-1	**1.78**
Spin-Orbit	19004	6-30-30-1	0.09

Supplementary Table 1. Number of ab initio points, parameters of NN structures and fitting errors of the new F+H_2O PES

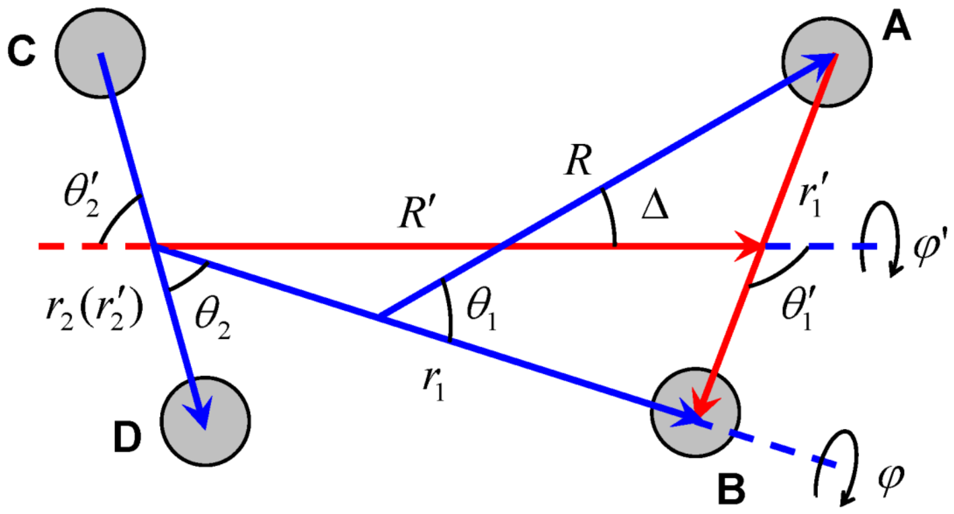

Supplementary Figure 1. The reagent Jacobi coordinates $(R, r_1, r_2, \theta_1, \theta_2, \varphi)$ for the A+BCD atom-triatom arrangement and product Jacobi coordinates $(R', r_1', r_2', \chi, \theta_1', \theta_2', \varphi')$ for the AB+CD diatom-diatom arrangement. Δ is the angle between $\hat{R}$ and $\hat{R}'$.

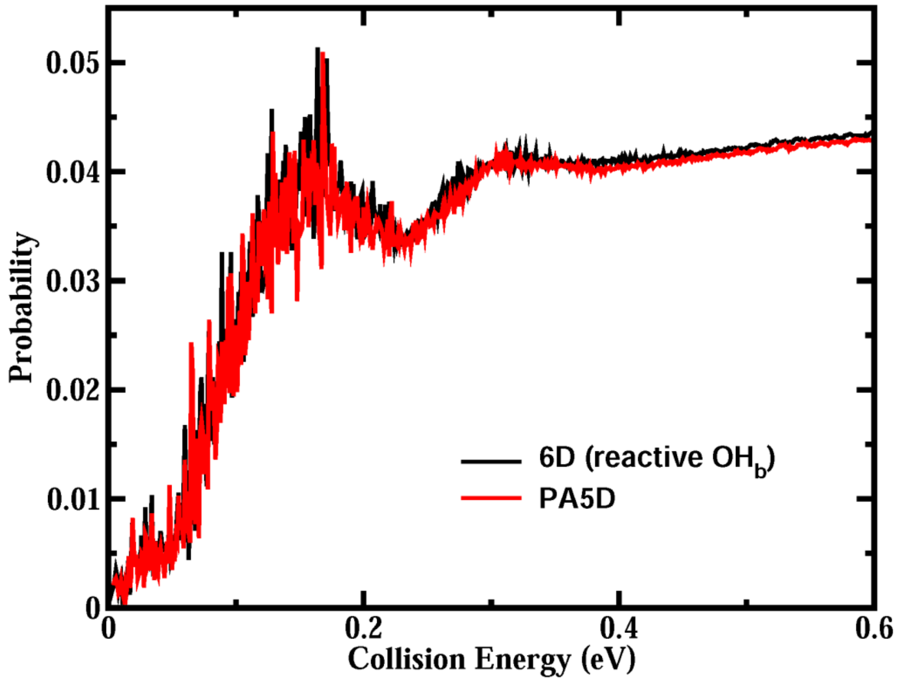

Supplementary Figure 2. The 6D and PA5D total reaction probabilities as a function of collision energy corresponding to the ground initial state.

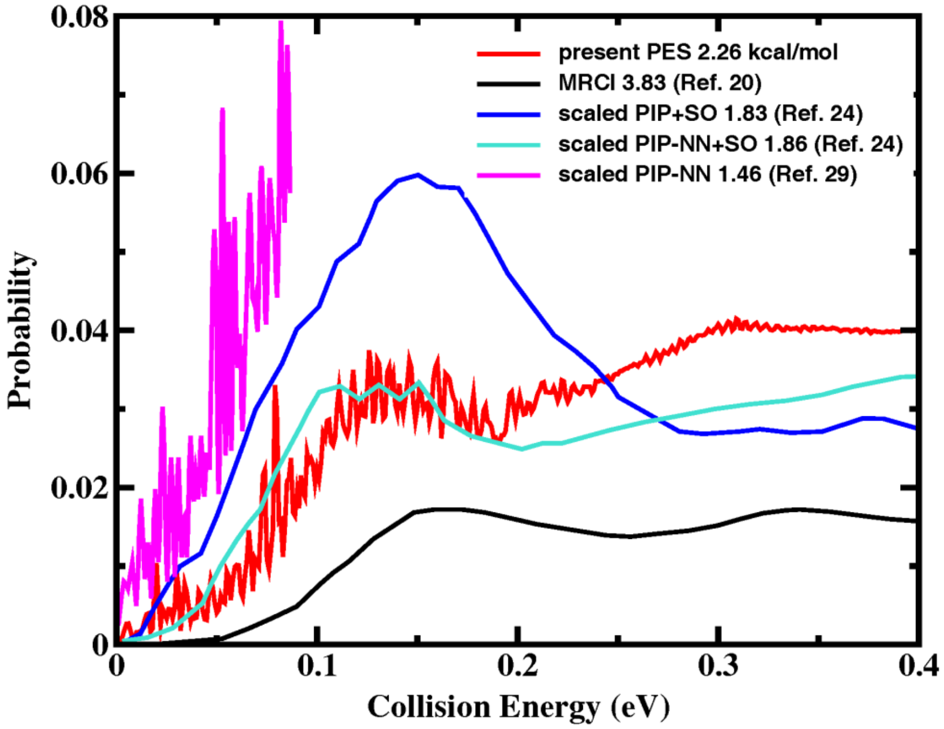

Supplementary Figure 3. The total reaction probabilities as a function of collision energy, in comparison with the QM results on the original MRCI PES and the scaled-MRCI ones by Guo and co-workers.

Supplementary References

[1] Hill, J. G., Mazumder, S. & Peterson, K. A. Correlation consistent basis sets for molecular core-valence effects with explicitly correlated wave functions: The atoms B–Ne and Al–Ar. *J. Chem. Phys.* **132**, 054108 (2010).

[2] Berning, A., Schweizer, M., Werner, H. –J., Knowles, P. J. & Palmieri, P.. Spin-orbit matrix elements for internally contracted multireference configuration interaction wavefunctions, *Molecular Physics*, **98**, 1823-1833(2000).

[3] Knowles, P. J., Werner, H. -J., Knizia, G., Manby, F. R., Schütz, M. et al. Molpro, version 2012.1, a package of ab initio programs (2012), see http://www.molpro.net.

[4] Chen, J. & Zhang, D. H. Construction of molecular reactive potential energy

surfaces based on neural networks. *Scientia Sinica Chimica* **45**, 1241 (2015).

5. Zhang, D. H. State-to-state quantum reactive scattering for four-atom chemical reactions: differential cross section for the $H+H_2O \rightarrow H_2+OH$ abstraction reaction. *J. Chem. Phys.* **125**, 133102 (2006).

6. Fu, B. & Zhang, D. H. A Time-Dependent Quantum Dynamical Study of the H + HBr Reaction. *J. Phys. Chem. A* **111**, 9516-9521 (2007).
